# RNA N^6^-Methyladenine Modification, Cellular Reprogramming, and Cancer Stemness

**DOI:** 10.3389/fcell.2022.935224

**Published:** 2022-07-04

**Authors:** Huarong Chen, Yifei Wang, Hao Su, Xiaoting Zhang, Hongyan Chen, Jun Yu

**Affiliations:** ^1^ Department of Anaesthesia and Intensive Care and Peter Hung Pain Research Institute, The Chinese University of Hong Kong, Hong Kong, China; ^2^ Institute of Digestive Disease and Department of Medicine and Therapeutics, State Key Laboratory of Digestive Disease, Li Ka Shing Institute of Health Sciences, CUHK Shenzhen Research Institute, The Chinese University of Hong Kong, Hong Kong, China; ^3^ Department of Anatomical and Cellular Pathology, State Key Laboratory of Oncology in South China, Prince of Wales Hospital, The Chinese University of Hong Kong, Hong Kong, China

**Keywords:** RNA metabolism, stemness, tumorigenesis, N^6^-methyladenose, reprogramming

## Abstract

N^6^-Methyladenosine (m^6^A) is the most abundant modification on eukaryote messenger RNA and plays a key role in posttranscriptional regulation of RNA metabolism including splicing, intracellular transport, degradation, and translation. m^6^A is dynamically regulated by methyltransferases (writers), RNA-binding proteins (readers), and demethylases (erasers). Recent studies demonstrate that perturbation of m^6^A regulators remarkably influences cell fate transitions through rewiring various biological processes, such as growth, differentiation, and survival. Moreover, aberrant m^6^A modification is implicated in a variety of diseases, in particular cancer. In this review, we describe the functional linkage of m^6^A modifications to cellular reprogramming and cancer stemness properties.

## Introduction

N^6^-Methyladenosine modification (m^6^A) refers to the methylation of the adenosine base at the nitrogen-6 position and tends to occur in a consensus sequence RRACH. It was originally discovered in 1970s and now recognized as the most abundant modification present in eukaryotic messenger RNA (mRNA) (Desrosiers et al., 1974; Adams and Cory, 1975; Lavi and Shatkin, 1975; Wei et al., 1975). m^6^A modification is present in different types of RNAs including mRNAs, transfer RNAs (tRNAs), ribosomal RNAs (rRNAs), circular RNAs (circRNAs), micro RNAs (miRNA), and long non-coding RNAs (lncRNAs) (Liu and Pan, 2016). The process of m^6^A modification is reversible and regulated by methyltransferases (writers), demethylases (erasers), and RNA-binding proteins (readers). Methyltransferase complex consisting of methyltransferase 3 (METTL3) (Bokar et al., 1997), methyltransferase 14 (METTL14) (Liu et al., 2014), and WT1-associated protein (WTAP) (Ping et al., 2014) catalyzes m^6^A formation. Other m^6^A writers such as RNA-binding motif protein 15/15B (RBM15/15B) (Patil et al., 2016), vir-like M6A methyltransferase associated (VIRMA) (Yue et al., 2018), and zinc finger CCCH-type containing 13 (ZC3H13) (Wen et al., 2018) have been identified to facilitate the function of the methyltransferase complex. On the other hand, fat mass and obesity-associated protein (FTO) (Jia et al., 2011) and AlkB homolog H5 (ALKBH5) (Zheng et al., 2013; Alemu et al., 2016), two key demethylases, demethylate m^6^A modification. Besides, m^6^A readers, e.g., YTH domain-containing proteins (YTHDF1-3 (Wang et al., 2014a; Wang et al., 2015; Shi et al., 2017) and YTHDC1-2 (Xiao et al., 2016; Wojtas et al., 2017)) and insulin-like growth factor-2 mRNA-binding proteins (IGF2BP1/2/3) (Huang et al., 2018), target m^6^A marks of transcripts and trigger RNA processing and metabolism such as alternative splicing, intracellular transport, degradation, and translation.

Self-renewal and differentiation are two unique properties of stem cells with the former referring to the capability of stem cells to make more stem cells and maintain the undifferentiated state, while the latter indicating the change of stem cells to a more specialized cell type. Notably, the processes of self-renewal and differentiation are controlled at a transcriptional level wherein epigenetic and epitranscriptomic regulation play critical roles. To date, m^6^A is proven to be a mark of transcriptome flexibility involved in regulating the function of stem cells. Emerging evidence have demonstrated that m^6^A modifications are involved in the process of mouse embryonic development (Geula et al., 2015), stem cell self-renewal (Batista et al., 2014; Wang et al., 2014b), spermatogenesis (Zheng et al., 2013), and so on. However, the origins and functions of m^6^A marks in reprogramming stemness properties are still largely unclear.

Perturbation of m^6^A regulators strongly affects gene expression patterns and biological functions of cells, leading to a variety of diseases including cancer. Recent evidence reveals a subpopulation of tumor cells, named cancer stem cells (CSCs), responsible for tumor initiation, metastasis, and relapse. The roles of cancer stem cells have been reported in both solid (Visvader and Lindeman, 2008) and hematological cancers (Zagozdzon and Golab, 2015), although the origin of the CSCs remains elusive. They may derive from differentiated cells or tissue-resident stem cells upon tumor initiation. Intriguingly, genes critical for self-renewal of normal stem cells also function as cancer-related genes, e.g., Bmi-1 (Siddique and Saleem, 2012), Nanog (Gawlik-Rzemieniewska and Bednarek, 2016), Notch (Ranganathan et al., 2011), Sox2 (Novak et al., 2020), and Wnt (Zhan et al., 2017). Given that m^6^A modifications regulate the expression of stemness-related genes, it is not surprising that they also play an important role in CSCs (Zhang et al., 2016; Li et al., 2017; Zhang et al., 2017; Chen et al., 2021).

In this review, we discuss recent studies that underscore the multifaceted role of m^6^A modifications in controlling gene expression, highlighting key findings that m^6^A modifications are essential in stem cells reprogramming and cancer stemness properties regulation.

## N^6^-Methyladenosine and RNA Metabolism

**FIGURE 1:**
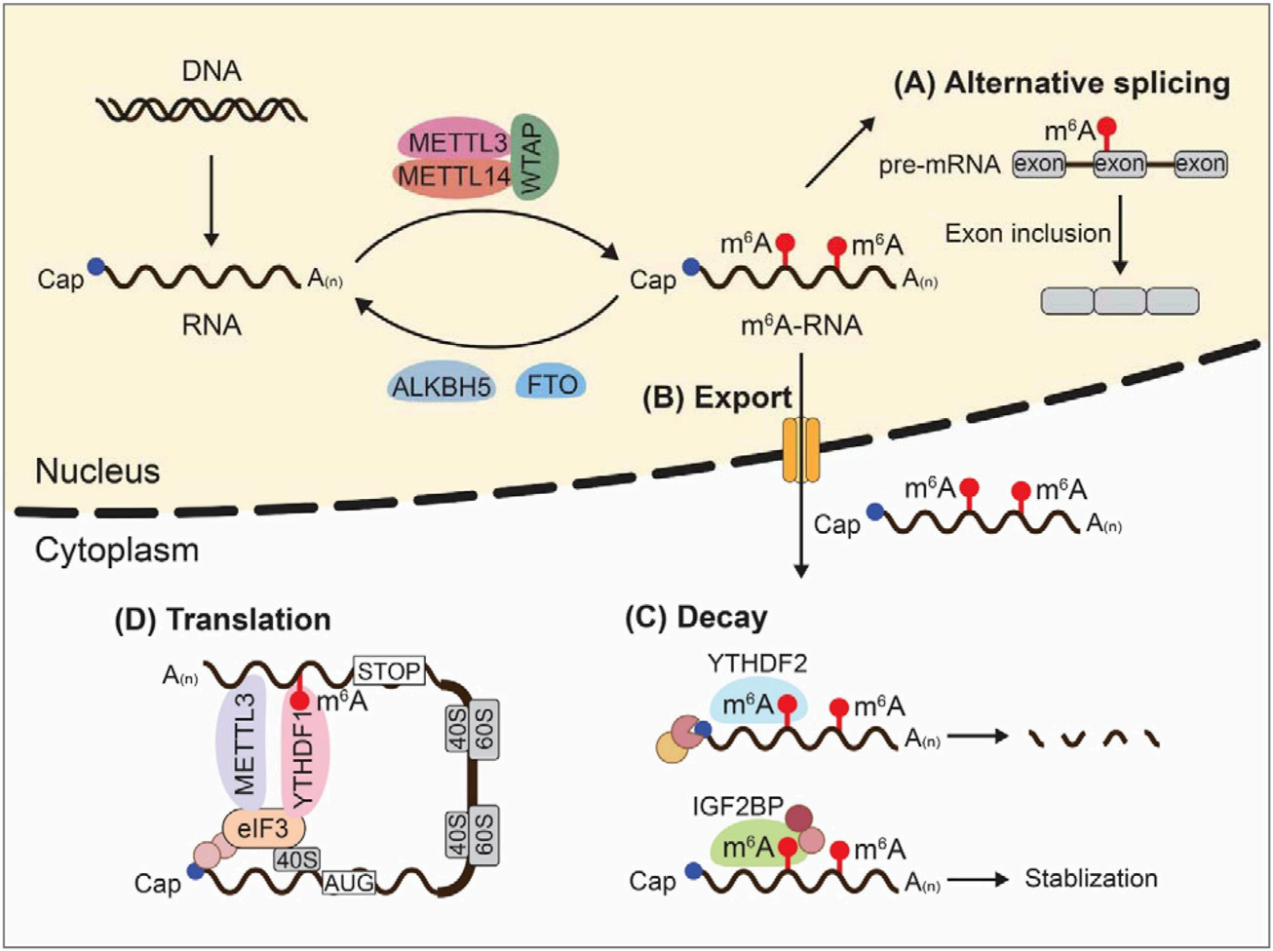
m^6^A modifications and RNA metabolism. Dynamic m^6^A modifications inside cells influence almost every step of RNA metabolism including alternative splicing, intracellular transport, degradation, and translation.

m^6^A controls almost every step of RNA metabolism including alternative splicing, intracellular transport, degradation, and translation (Figure 1). In this part, we describe the influences of m^6^A writers, erasers, and readers on RNA metabolic process through dynamic regulation of m^6^A.

### N^6^-Methyladenosine and Alternative Splicing

Alternative splicing (AS) is the process of making messenger RNA (mRNA) from messenger RNA precursor (pre-mRNA) by selecting different combinations of splice sites in pre-mRNA, thus allowing a single gene to code for multiple proteins. AS is essential for generating functional diversity given the limited gene number in eukaryotic organisms. Emerging evidence shows that m^6^A writers, METTL3, METTL14, and WTAP, and m^6^A erasers, ALKBH5 and FTO, are located in nuclear speckles where the AS occurs, indicating a potential role of m^6^A for controlling pre-mRNA processing. In support of this, treatment of S-adenosylmethionine (SAM) synthesis inhibitors, neplanocin A or cycloleucine, reduced RNA m^6^A methylation and resulted in nuclear accumulation of unspliced transcripts (Stoltzfus and Dane, 1982; Carroll et al., 1990). Consistently, Dominissini et al. observed a correlation between m^6^A methylation of multi-isoform genes and isoform switching by analyzing human and mouse transcriptome-wide m^6^A profiling (Dominissini et al., 2012).

METTL3 was found to colocalize with spliceosomal protein U2 small nuclear ribonucleoprotein B'' (U2B′)' in nuclear speckles (Bokar et al., 1997). Silencing of METTL3 could affect AS patterns and gene expressions (Dominissini et al., 2012). WTAP is a regulatory subunit of RNA m^6^A methyltransferase complex. Localization of METTL3 and METTL14 into nuclear speckles requires interaction with WTAP. Yang et al. showed that WTAP promoted METTL3 and METTL14 accumulation in nuclear speckles and regulated AS of targeted genes (Ping et al., 2014). Knockdown of METTL3 or WTAP led to a remarkable change of transcriptional isoform numbers (Ping et al., 2014). ALKBH5 also colocalized with mRNA-processing factors, including phosphorylated serine/arginine-rich splicing factor 2 (SC35), smith antigen (SM), and alternative splicing factor/splicing factor 2 (ASF/SF2) in nuclear speckles (Zheng et al., 2013). Similarly, FTO was present in nucleoplasm and partially colocalized with splicing factors U4/U6.U5 tri-snRNP-associated protein 1 (SART1), serine/arginine-rich splicing factor 2 (SC35), and RNA polymerase II phosphorylated at Ser2 (Pol II-S2P) (Jia et al., 2011). These two key m^6^A erasers are both capable of controlling mRNA splicing. ALKBH5 was found to regulate assembly of mRNA processing factors (Zheng et al., 2013); on the other hand, FTO depletion increased the m^6^A level of target genes, thereby raising the binding capacity of serine/arginine-rich splicing factor 2 (SRSF2) which subsequently increased inclusion of target exons (Zhao et al., 2014). m^6^A reader YTHDC1 is also engaged in the AS process. YTHDC1 was present in YT bodies near nuclear speckles. Wild type YTHDC1, but not m^6^A-binding-defective YTHDC1, could recruit pre-mRNA splicing factor SRSF3 (SRp20) but block the binding of SRSF10 (SRp38) to targeted mRNAs in the nucleus, thus promoting exon inclusion (Xiao et al., 2016). All these data indicate an essential regulatory role of m^6^A in mRNA splicing.

### N^6^-Methyladenosine and RNA Export

RNAs produced in the nucleus are exported to the cytoplasm through nuclear pore complexes. This is a fundamental step in gene expression process. TREX complex is important for mRNA export. Recent work identified the interactions between TREX subunits (ALYREF, UAP56, THOC5, and CHTOP) and m^6^A methyltransferases (METTL3, METTL14, WTAP, and KIAA1429) (Lesbirel et al., 2018). The m^6^A methyltransferase complex could recruit TREX to m^6^A-modified mRNAs to facilitate their export (Lesbirel et al., 2018). Moreover, depletion of KIAA1429 and WTAP led to an export block for m^6^A-modified mRNAs (Lesbirel et al., 2018). m^6^A eraser ALKBH5 also affects mRNA export dependent on its demethylation activity. Cytoplasmic mRNA level was significantly increased after silencing of ALKBH5 because of accelerated nuclear RNA export; re-expression of wild type ALKBH5, but not catalytic inactive mutant H204A, could rescue this phenomenon (Zheng et al., 2013). Binding of YTHDC1 to m^6^A-modified genes is important for mRNA export. Knockdown of YTHDC1 induced an export block for nuclear m^6^A-modified mRNA, resulting in accumulation of transcripts in the nucleus (Roundtree et al., 2017). Mechanistically, YTHDC1 interacted with SRSF3, an mRNA export adaptor, to increase RNA binding to SRSF3 (Roundtree et al., 2017).

m^6^A modifications also participate in circular RNA nuclear export. Depletion of m^6^A writer METTL3 induced circNSUN2 accumulation in the nucleus, and re-expression of METTL3 could rescue this phenomenon (Chen et al., 2019). Moreover, the m^6^A reader YTHDC1 was capable of binding to m^6^A marks of circNSUN2 in the backsplicing junction sites to facilitate the export process (Chen et al., 2019). Together, m^6^A modifications regulate RNA export.

### N^6^-Methyladenosine and RNA Decay

RNA decay is the process whereby RNA is enzymatically degraded. RNA decay is important for effective mRNA surveillance and turnover. Accumulating evidence suggest m^6^A modifications affect RNA stability through dynamic interplays with RNA-binding proteins. In mouse embryonic stem cells, m^6^A level was found to be negatively correlated with mRNA stability (Wang et al., 2014b). m^6^A writers METTL3 and METTL14 could form a stable heterodimer to catalyze m^6^A deposition on RNA. Downregulation of METTL3 and METTL14 reduced the m^6^A level of mRNA, resulting in more binding of human antigen R (HuR) to mRNA which in turn promoted mRNA stability (Wang et al., 2014b). In line with these findings, depletion of METTL3 in both human and mouse cells led to m^6^A erasure and prolonged half-life of targeted mRNAs (Batista et al., 2014). Although WTAP lacks m^6^A catalytic activity, it binds to METTL3-METTL14 complex to enhance m^6^A deposition. As such, WTAP-mediated m^6^A modifications were negatively correlated with mRNA stability (Schwartz et al., 2014). Furthermore, silencing of METTL3, METTL14, or WTAP reduced global m^6^A methylation and increased the lifetime of nascent RNAs (Liu et al., 2014). Therefore, m^6^A modifications affect RNA stability.

Recent reports state that YTHDF2 is the major decay-inducing reader protein that binds to m^6^A-modified mRNAs to facilitate RNA degradation (Du et al., 2016; Park et al., 2019). Two distinct mechanisms of YTHDF2-induced mRNA degradation have been identified: RNase P/MRP-mediated endoribonucleolytic-cleavage pathway and carbon catabolite repression 4 (CCR4)-negative on TATA-less (NOT)-mediated deadenylation pathway, depending on whether messenger ribonucleoprotein (mRNP) has heat-responsive protein 12 (HRSP12)-binding site or not (Lee et al., 2020). Showed that m^6^A-modified RNAs underwent endoribonucleolytic cleavage *via* YTHDF2, HRSP12, and RNase P/MRP, of which HRSP12 acted as an adaptor to connect YTHDF2 and RNase P/MRP Park et al. (2019). In this case, HRSP12-binding site and RNase P/MRP-directed cleavage site were identified upstream and downstream of YTHDF2-binding site, respectively (Park et al., 2019). Of note, m^6^A-modified circular RNA could also be degraded through YTHDF2-HRSP12-RNase P/MRP-mediated endoribonucleolytic cleavage (Park et al., 2019). On the other hand, Du et al. reported that YTHDF2 directly recruited CCR4/NOT deadenylase complex to m^6^A-modified mRNAs, leading to deadenylation of mRNAs (Du et al., 2016). Besides, YTHDF3 was identified to regulate the RNA accessibility of YTHDF2 and enhanced YTHDF2-mediated mRNA decay (Shi et al., 2017). In contrast to YTHDF2-mediated mRNA decay, a recent study revealed that IGF2BP1-3 could recognize m^6^A markers through their KH domains to stabilize m^6^A-modified RNA (Huang et al., 2018). Intriguingly, although YTHDF2 and IGF2BP1-3 were all proved to bind to m^6^A markers, their transcriptome-wide binding sites were distinct (Huang et al., 2018). Therefore, m^6^A modifications can either enhance or inhibit mRNA stability depending on the binding of specific m^6^A readers.

### N^6^-Methyladenosine and Messenger RNA Translation

Translation is the decoding of mRNA by ribosomes to produce polypeptide which later forms a functional protein inside the cells. Recent studies demonstrate that m^6^A modifications modulate mRNA translation efficiency through different mechanisms. YTHDF1 is known to promote the translation of m^6^A-modified mRNA. Mechanistically, YTHDF1 could promote ribosome occupancy of targeted mRNA in the cytoplasm by recruiting the initiation factor eukaryotic initiation factor 3 (eIF3) (Wang et al., 2015). In addition, YTHDF3 was reported to facilitate YTHDF1-promoted translation (Shi et al., 2017). METTL3 also enhances mRNA translation. Barbieri et al. found that the transcription factor, CEBPZ, recruited METTL3 to the promoters of select active gene to catalyze m^6^A methylation in the coding region (CDS) of targeted mRNA, resulting in enhanced translation by relieving ribosome stalling (Barbieri et al., 2017). Consistently, knockdown of METTL3 decreased translational efficiency of m^6^A-modified transcripts in both human myeloid leukemia and HeLa cell lines (Vu et al., 2017). Surprisingly, METTL3-promoted translation could be independent of m^6^A catalytic activity (Lin et al., 2016). Gregory and others showed that tethering a wild type or catalytically inactive METTL3 to the 3′UTR of a reporter mRNA exhibited similar translation enhancement (Lin et al., 2016). They further identified a direct physical and functional interaction between METTL3 at 3′UTR near the stop codon and eIF3h at the 5′ untranslated region (5′ UTR) of the mRNA and that METTL3-eIF3h loop may promote translation through ribosome recycling (Choe et al., 2018). Intriguingly, depletion of YTHDF1 did not influence the expression of METTL3 targets (Choe et al., 2018). Thus, METTL3 promotes mRNA translation through diverse mechanisms. It is worth noting that mouse embryonic stem cells (mESCs) with METTL3 knockout exhibited a modest increased translation efficiency (TE) compared to wild type (WT) cells, although this effect was observed for both methylated and unmethylated transcripts with higher GC content (Geula et al., 2015). In this study, loss of m^6^A could directly enhance mRNA stability of m^6^A-marked transcripts while indirectly favoring translation of GC-rich transcripts (Geula et al., 2015). Intriguingly, Slobodin et al. reported that transcription rate positively affected the efficiency of mRNA translation which was mediated by m^6^A modification (Slobodin et al., 2017). Therefore, mRNA m^6^A could mediate the communication between transcription and translation.

Qian and Jaffrey’s team suggested that m^6^A could enable mRNA translation in a cap- and IRES-independent manner (Meyer et al., 2015; Zhou et al., 2015; Coots et al., 2017; Zhou et al., 2018). They showed that heat shock stress promoted nuclear localization of YTHDF2 which in turn increased 5′ UTR m^6^A of stress-inducible mRNAs through competing with FTO in preserving m^6^A modification, leading to enhanced cap-independent translation initiation (Zhou et al., 2015). In addition, eIF3 could bind to 5′ UTR m^6^A and recruit the 43S complex to initiate translation without the cap-binding factor eIF4E under stress (Meyer et al., 2015). Furthermore, depletion of METTL3 selectively inhibited translation of mRNAs with 5′ UTR m^6^A, but not mRNAs with 5′ terminal oligopyrimidine (TOP) elements (Coots et al., 2017). Notably, ABCF1 was identified to coordinate with METTL3 in promoting translation of m^6^A-modified mRNA (Coots et al., 2017). Thus, 5′ UTR m^6^A facilitates cap-independent translation under stress.

m^6^A is also thought to facilitate efficient translation of circular RNA (circRNA) (Yang et al., 2017). Initiation factor eIF4G2 and YTHDF3 were identified to be required for m^6^A-driven circRNAs translation, which were enhanced by METTL3/14-mediated methylation or suppressed by FTO-mediated demethylation (Yang et al., 2017). Consistently, Bozzoni et al. demonstrated that METTL3 and YTHDC1 could direct the back-splicing reaction of circRNAs, and recognition of m^6^A marks by YTHDF3 and eIF4G2 modulate circRNAs translation (Di Timoteo et al., 2020).

## N^6^-Methyladenosine and Cellular Reprogramming

Mammalian development is thought to be continuous and unidirectional in which stem cells give rise to specialized differentiated cells through a series of cellular changes. However, recent studies have shown that it is possible to modify cell identity by somatic cell nuclear transfer (SCNT) (Matoba and Zhang, 2018), forced expression of specific transcription factors (Takahashi and Yamanaka, 2016) or micro-RNAs (Judson et al., 2009), and using small signaling molecules (Hou et al., 2013). In 2006, Kazutoshi Takahashi and Shinya Yamanaka successfully reprogrammed mouse embryonic fibroblasts (MEF) and adult mouse tail-tip fibroblasts to generate induced pluripotent stem cells (iPSCs) by ectopic expression of four transcription factors, namely Oct3/4, Sox2, c-Myc, and Klf4 (Takahashi and Yamanaka, 2006). In 2007, they further demonstrated the generation of iPSC from adult human dermal fibroblasts with the same four factors (Takahashi et al., 2007). The fact that terminally differentiated somatic cells can be reprogrammed to generate iPSCs has opened new gateways for therapeutics research. Recent evidence has revealed epigenetic profile changes during the process of cell differentiation and reprogramming and that epigenetic perturbations could affect the efficiency of reprogramming iPSCs (Young, 2011; Liang and Zhang, 2013; Hochedlinger and Jaenisch, 2015; Xu and Xie, 2018). In this part, we describe the influences of m^6^A modifications on stemness and reprogramming.

### N^6^-Methyladenosine and Stemness

To maintain self-renewal and pluripotency, stem cells need to stably express pluripotency genes; however, they are also capable of rapidly altering gene expression programming for differentiation. m^6^A is involved in cell fate determination and is now considered as a mark of transcriptome flexibility required by stem cells. Zhao and others identified that depletion of METTL3 or METTL14 in mESCs suppressed m^6^A methylation and self-renewal capability (Wang et al., 2014b). Mechanistically, m^6^A marks blocked the binding of RNA stabilizer protein HuR and protected mRNA from degradation induced by RNA-induced silencing complex (RISC) (Wang et al., 2014b). Consequently, developmental regulators were more enriched than pluripotency genes upon METTL3 or METTL14 knockdown (Wang et al., 2014b). Thus, METTL3/METTL14-mediated m^6^A modification is required to maintain the pluripotency of ES cells. However, Batista et al. reported that m^6^A loss promoted ESC self-renewal and hindered differentiation (Batista et al., 2014). In this study, they profiled m^6^A methylome in mouse and human ESCs, revealing extensive m^6^A modification of ESC genes, including core pluripotency regulators such as Nanog, Klf4, Myc, Lin28, Med1, Jarid2, and Eed (Batista et al., 2014). They considered m^6^A as a mark for RNA turnover over in a timely fashion, and knockout of METTL3 improved mESCs self-renewal without affecting cell viability (Batista et al., 2014). The differences in phenotypes between these two studies may partially be explained by the methodology used (RNAi and CRISPR) which may affect downstream m^6^A-modified RNAs pattern. Another possibility is that the mESCs used in these two studies were at different states. TGFβ signaling is essential for human pluripotent stem cells (hPSCs) to maintain pluripotency (James et al., 2005). Vallier et al. identified a functional interaction between SMAD2/3 transcription factors and METTL3-METTL14-WTAP complex (Bertero et al., 2018). SMAD2/3 could promote the binding of METTL3-METTL14-WTAP to specific SMAD2/3 transcriptional targets involved in early cell fate decisions, e.g., pluripotency factor gene NANOG, leading to increased m^6^A methylations that facilitate mRNA degradation (Bertero et al., 2018). Consequently, m^6^A-mediated rapid downregulation of SMAD2/3-targeted genes facilitated timely shut down of pluripotency on differentiation (Bertero et al., 2018). Intriguingly, Filipczyk and others reported that depletion of m^6^A could both support pluripotency maintenance and exit through activating pAkt and pErk signaling, respectively (Jin et al., 2021).

m^6^A modification is required for embryo development. Knockout of METTL3 or METTL14 led to early embryonic lethality (Geula et al., 2015; Meng et al., 2019). In *Mettl3* ^*−/−*^ mice, preimplantation epiblasts and naïve embryonic stem cells with loss of m^6^A were still viable; however, they failed to terminate the naïve state toward lineage differentiation, resulting in early embryonic lethality (Geula et al., 2015). The abnormal expression and location of NANOG caused by METTL3 ablation was regarded as the leading cause (Geula et al., 2015). Meanwhile, METTL14 is indispensable for postimplantation embryonic development. Silencing of METTL14 contributed to abnormal embryo development since embryonic day 6.5 (E6.5), mainly due to resistance to differentiation (Meng et al., 2019). Mechanistically, METTL14 depletion caused dysregulation of genes associated with embryo development pathways (Meng et al., 2019). The m^6^A readers YTHDF2 and YTHDC1 are also important for mammalian development (Ivanova et al., 2017; Kasowitz et al., 2018). Maternal RNA degradation, which was mediated by YTHDF2, facilitated oocyte maturation; oocytes with YTHDF2 deficiency failed to change metaphase II (MII) transcriptome, leading to female-specific infertility in mice (Ivanova et al., 2017). On the other hand, knockout of YTHDC1 caused massive alternative splicing defects in oocytes, resulting in a block at the primary stage of folliculogenesis (Kasowitz et al., 2018).

### N^6^-Methyladenosine and Epitranscriptomic Reprogramming

The epigenetic modifications could lock cells into a differentiated state during cell differentiation; therefore, targeting repressive epigenetic marks in differentiated cells improve the efficiency of iPSC formation (Huangfu et al., 2008; Shi et al., 2008). Recent studies also pinpoint m^6^A as an important player during cellular reprogramming. Chen et al. reported that m^6^A formation facilitated cell reprogramming to pluripotency (Chen et al., 2015). In this study, lots of cell-type specific markers are m^6^A-modifed, such as Oct4, Nanog, and DPPA2 for ESCs and iPSCs; POU3F2 and ROBO2 for neural stem cells; and DHH and Sox8 for testicular sertoli cells (Chen et al., 2015). These genes are critical for stem cell maintenance and developmental regulation. Intriguingly, miRNAs could target m^6^A marks by base pairing and modulate the binding of METTL3, thus leading to the change of cellular m^6^A abundance (Chen et al., 2015). Deletion of Dicer, an essential endonuclease for producing mature miRNAs, remarkably inhibited the RNA m^6^A level; in contrast, overexpression of miRNAs increased the binding of METTL3 on mRNAs and enhanced m^6^A abundance (Chen et al., 2015). To investigate the role of m^6^A in cell reprogramming, manipulation of METTL3 was conducted in MEFs transduced with four Yamanaka transcription factors. The result indicated that ectopic expression of METTL3 increased colonies of iPSC, enhanced expressions of key pluripotent factors (Oct4, Sox2, and Nanog), and promoted the reprogramming of MEFs to pluripotent stem cells; conversely, depletion of METTL3 reduced m^6^A and led to impeded reprogramming (Chen et al., 2015).

The crosstalk between epigenetic and epitranscriptomic networks is important to cellular reprogramming. Aguilo et al. reported that chromatin-associated zinc finger protein 217 (ZFP217) coordinated epigenetic and epitranscriptomic regulation to ensure ESC self-renewal and somatic cell reprogramming (Aguilo et al., 2015). They identified gradually increased ZFP217 expression along with decreased METTL3 expression during somatic reprogramming (Aguilo et al., 2015). ZFP217 could induce transcription of core reprogramming factors and repress m^6^A deposition of pluripotency genes by sequestering METTL3 (Aguilo et al., 2015). Depletion of ZFP217 in MEFs increased the m^6^A level of Nanog, Sox2, Klf4, and c-Myc mRNAs, promoting their degradation and leading to diminished iPSC colonies formation; this phenomenon could be partially rescued by METTL3 knockdown (Aguilo et al., 2015). Therefore, m^6^A modifications may be a barrier for ZFP217-meidiated somatic cell reprogramming. In support of these, Song et al. demonstrated that ZFP217 suppressed m^6^A mRNA methylation by promoting FTO expression (Song et al., 2019). Silencing of ZFP217 decreased FTO expression to enhance m^6^A levels, resulting in retarded adipogenic differentiation (Song et al., 2019).

So, how to understand the conflicting phenomena regarding the role of m^6^A on somatic cell reprogramming? One possible explanation is that m^6^A on cell fate choice is context dependent. Geula et al. reported that depletion of METTL3 exerted a divergent effect on naïve and primed PSCs (Geula et al., 2015). In naïve PSCs, pluripotency genes were highly expressed, and silencing of METTL3 could further enhance their expression to boost naïve circuitry stability; by contrast, the expression of pluripotency genes was downregulated while lineage commitment markers were upregulated in primed cells; thus silencing of METTL3 exerted a minor effect on expression of pluripotency genes while it remarkably increased the expression of lineage commitment markers, making the cells tend toward differentiation (Geula et al., 2015). Therefore, epigenetics and epitranscriptomics can form a complex network to regulate stem cell pluripotency and differentiation.

## N^6^-Methyladenosine and Cancer Stem Cells

**FIGURE 2:**
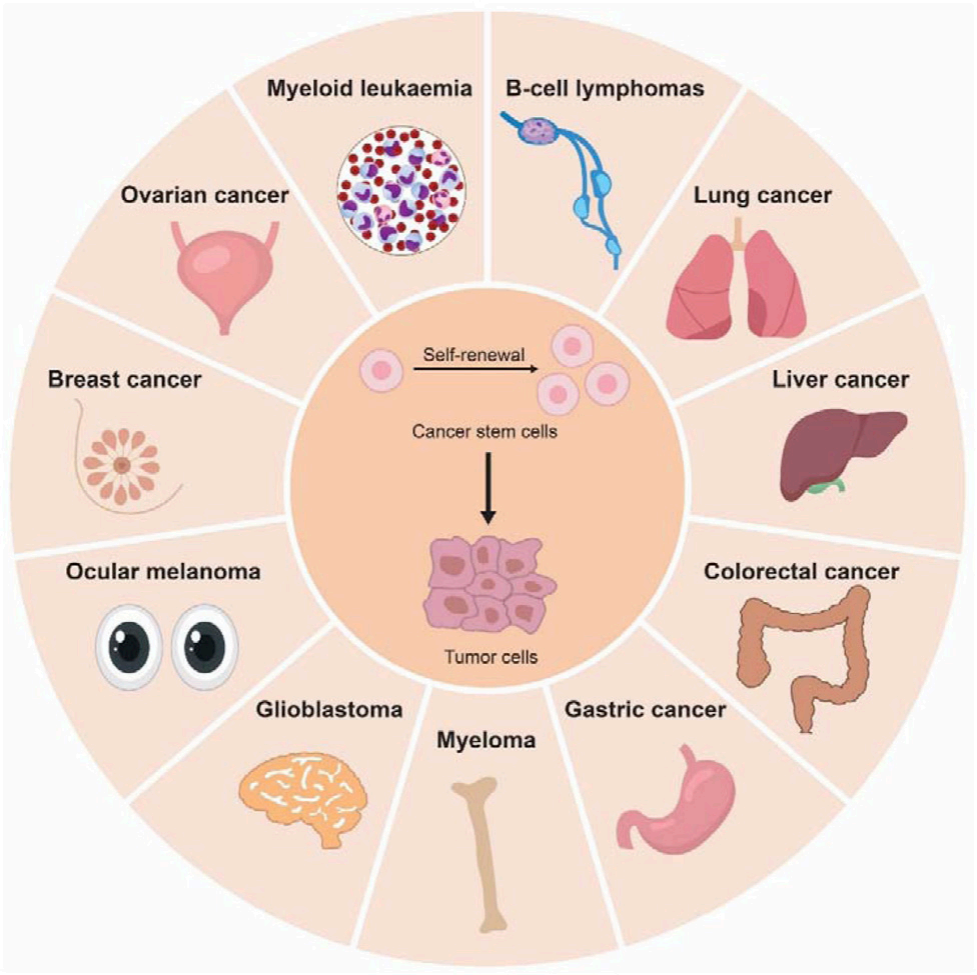
Cancer stem cells are key drivers to tumor initiation and progression. Cancer stem cells (CSCs) have been identified in different type of solid cancers (colorectal cancer, gastric cancer, liver cancer, glioblastoma, melanoma, breast cancer, lung cancer, and ovarian cancer) and hematological cancers (myeloid leukemia, lymphoma, and myeloma).

CSCs or tumor-initiating cells (TICs) are a small subpopulation of cancer cells which could give rise to tumors through processes of self-renewal and differentiation, just like normal stem cell (Figure 2). Tumor development and iPSC generation share striking similarities on gene expression programming, implying a potential link between pluripotency and cancer (Wong et al., 2008). Furthermore, recent evidence state that cancer cells could be reprogrammed to retrieve benign cell functions or differentiate into other unrelated cell types by re-expression of lineage-specific genes, opening a new avenue for cancer treatment (Bussard et al., 2010; Pezzolo et al., 2011). Understanding the molecular drivers of CSCs will advance the development of anticancer therapeutics. In this section, we summarize the key findings on how m^6^A modifications modulate cancer stemness (Table 1).

**TABLE 1:** M^6^A-mediated molecular events in different cancer types.

Cancer type	Molecular event
Acute myeloid leukemia	FTO-m^6^A-ASB2/RARA Li et al. (2017)
	FTO-m^6^A-MYC/CEBPA Su et al. (2018)
Breast cancer	ALKBH5-m^6^A-NANOG Zhang et al. (2016)
	METTL14-m^6^A-DROSHA Peng et al. 2021)
Colorectal cancer	METTL3-m^6^A-GLUT1-mTORC1 Chen et al. (2021)
	YTHDF1-m^6^A-ARHGEF2 Wang et al. (2022)
	YTHDF1-m^6^A-TCF7L2/TCF4-β-catenin Han et al. (2020)
Gastric cancer	YTHDF1-m^6^A-FZD7-Wnt/β-catenin Pi et al. (2021)
Glioblastoma	YTHDF2-m^6^A-MYC-IGFBP3 Dixit et al. (2021)
	ALKBH5-m^6^A-FOXM1 Zhang et al. (2017)
	METTL3/METTL14-m^6^A-ADAM19 Cui et al. (2017)
	METTL3-m^6^A-SRSF Li et al. (2019)
Liver cancer	YTHDF2-m^6^A-OCT4 Zhang et al. (2020)
	METTL14-m^6^A-HNF3γ Zhou et al. (2020)
	FTO-m^6^A-TGF-β2 Zhou et al. (2020)
Lung cancer	ALKBH5-m^6^A-Nanog/Oct4 Liu et al. (2022)
Lymphoma	WTAP-m^6^A-HK2 Han et al. (2021)
	ALKBH5/FTO-m^6^A-SPI1/PHF12 Wu et al. (2021)
Multiple myeloma	FTO-m^6^A-HSF1 Xu et al. (2022)
	ALKBH5-m^6^A-TRAF1 Qu et al. (2022)
Ovarian cancer	FTO-m^6^A-PDE1C/PDE4B Huang et al. (2020)

### N^6^-Methyladenosine and Solid Tumors

Solid tumors refer to an abnormal mass of tissue in “solid” organs. Gastrointestinal (GI) cancer is one of them, referring to cancers that affect the digestive system, e.g., colorectal cancer (CRC), gastric cancer (GC), and liver cancer. Several m^6^A regulators have been reported to play important roles in GI cancer. Our team recently identified the novel oncogenic epitranscriptome axis of METTL3-m^6^A-GLUT1-mTORC1 (Chen et al., 2021) and YTHDF1-m^6^A-ARHGEF2 (Wang et al., 2022) in promoting CRC tumorigenesis. In the former study, METTL3 was found to promote GLUT1 translation in an m^6^A-dependent manner by integrative m^6^A sequencing, RNA sequencing, and ribosome profiling analyses, resulting in increased glucose uptake and lactate production which subsequently activated mTORC1 signaling; consequently, depletion of METTL3 impaired the self-renewal capacity of colon cancer-initiating cells (Chen et al., 2021). As to the latter study, knockdown of YTHDF1 suppressed CRC organoids and decreased cell growth; mechanistically, YTHDF1 bind to m^6^A marks of ARHGEF2 mRNA and enhanced ARHGEF2 translation by multiomic analysis of m^6^A sequencing, RNA sequencing, YTHDF1 RNA immunoprecipitation sequencing and proteomics (Wang et al., 2022). In line with our findings, Han et al. reported that high expression of YTHDF1 was induced by Wnt signaling in intestinal stem cells (ISCs) which in turn promoted translation of TCF7L2/TCF4, leading to enhanced β-catenin activity that promoted stemness of ISCs (Han et al., 2020). YTHDF1 also activates Wnt/β-catenin signaling in GC. Pi et al. revealed that YTHDF1 increased translation of frizzled7 (FZD7), a key Wnt receptor, in an m^6^A-dependent manner; consequently, hyperactivation of the Wnt/β-catenin was induced that facilitated GC tumorigenesis (Pi et al., 2021). Intriguingly, long non-coding RNAs (lncRNAs) could influence the m^6^A modification process. LNC942 was identified to induce GC stemness and chemoresistance by stabilizing Musashi2 (MSI2), a member of RNA-binding proteins (RBPs); MSI2 then bind to m^6^A sites of c-Myc mRNA to increase mRNA stability (Zhu et al., 2022).

Current evidence also pinpoints a pivotal role of m^6^A modifications in liver cancer stem cells (Zhang et al., 2020; Zhou et al., 2020; Bian et al., 2021; Wang et al., 2021). In purified CD133^+^ liver cancer stem cells, knockdown of YTHDF2 impaired tumor-initiating ability; in contrast, overexpression of YTHDF2 exerted the opposite effect (Zhang et al., 2020). YTHDF2 was capable of binding to m^6^A sites in the 5′UTR of OCT4 mRNA to promote its translation as determined by luciferase activity assay and polysome profiling assay (Zhang et al., 2020). Meanwhile, METTL14 induced m^6^A methylation of hepatocyte nuclear factor 3γ (HNF3γ) mRNA, a hepatocyte nuclear factor, leading to reduced HNF3γ expression in hepatocellular carcinoma (HCC) (Zhou et al., 2020). Notably, enforced HNF3γ expression promoted differentiation of HCC cells and liver CSCs, resulting in retarded growth of HCC (Zhou et al., 2020). In addition, HNF3γ expression rendered sensitivity of HCC cells to sorafenib treatment, implying the potential of HNF3γ as a therapeutic target for HCC (Zhou et al., 2020). RALY RNA-binding protein-like (RALYL), a liver progenitor specific gene, was also related with HCC differentiation (Wang et al., 2021). Overexpression of RALYL suppressed the m^6^A level of TGF-β2 mRNA to enhance its mRNA stability, leading to subsequent activation of TGF-β signaling that contributed to HCC self-renewal and chemoresistance (Wang et al., 2021). In this study, FTO was found to bind to RALYL and thought to be responsible for m^6^A demethylation of TGF-β2 mRNA (Wang et al., 2021). Furthermore, FTO-mediated RNA demethylation was also involved in S-adenosylmethionine decarboxylase proenzyme (AMD1)-induced cancer stemness in HCC (Bian et al., 2021). AMD1 was capable of stabilizing the interaction between Ras GTPase-activating-like protein 1 (IQGAP1) and FTO, leading to enhanced FTO expression which in turn promoted HCC stemness (Bian et al., 2021). Together, m^6^A modifications are critical for self-renewal and differentiation of CSCs in GI cancer.

Dysregulated m^6^A modifications play an important role in lung cancer. Yin et al. identified an lncRNA named RNA Component of Mitochondrial RNA Processing Endoribonuclease (RMRP) which exhibited enriched m^6^A modifications and increased RNA stability in non-small cell lung cancer (NSCLC) (Yin et al., 2021). Both *in vitro* and *in vivo* experiments in this study revealed that RMRP induced TGFBR1/SMAD2/SMAD3 axis and promoted the cancer stem cell properties of NSCLC (Yin et al., 2021). However, how m^6^A modifications regulate RMRP stability warrants further investigation. On the other hand, Liu et al. (2022) recently reported that ALKBH5 was highly expressed in CSCs isolated from NSCLC. They revealed that depletion of ALKBH5 increased the global m^6^A level, suppressed expression of Nanog and Oct4, two essential transcription factors for self-renewal and pluripotency of ESCs, and inhibited stemness of CSCs (Liu et al., 2022). Intriguingly, p53 was reported to regulate malignancies of CSCs partially through transactivating ALKBH5 expression (Liu et al., 2022).

Glioblastoma is a prevalent and malignant cancer that occurs in the brain or spinal cord. m^6^A modifications could regulate gene expression and cell fate in glioblastoma stem-like cells (GSCs). Compared to normal neural stem cells (NSCs), GSCs preferentially expressed YTHDF2 which was essential for GSCs maintenance (Dixit et al., 2021). Intriguingly, instead of destabilizing mRNAs, YTHDF2 was found to increase MYC and VEGFA mRNA stability in an m^6^A-dependent manner in GSCs, although the mRNA-stabilizing function of YTHDF2 was unclear (Dixit et al., 2021). YTHDF2-MYC-IGFBP3 axis was further identified to promote glioblastoma growth both *in vitro* and *in vivo* (Dixit et al., 2021). Importantly, administration of linsitinib, an IGF1/IGF1R inhibitor, exerted potent inhibitory effect against YTHDF2-expressing GSCs without affecting NSCs (Dixit et al., 2021). ALKBH5 was also found highly expressed in GSCs, and silencing of ALKBH5 inhibited the growth of patient-derived GSCs (Zhang et al., 2017). Mechanistically, ALKBH5 reduced the m^6^A level of FOXM1 mRNA, resulting in enhanced FOXM1 expression which in turn promoted GSC tumorigenesis (Zhang et al., 2017). m^6^A modifications are critical for self-renewal of GSCs. Knockdown of METTL3 or METTL14 promoted growth, self-renewal, and tumorigenesis of human GSC; conversely, overexpression of METTL3 or inhibition of FTO exerted the opposite effect (Cui et al., 2017). ADAM19 was a downstream target of METTL3/METTL14 that exerted critical biological functions in GSCs (Cui et al., 2017). m^6^A modifications could influence nonsense-mediated mRNA decay (NMD) in GSCs. Li et al. reported that METTL3 regulated the NMD of splicing factors and AS process in glioblastoma (Li et al., 2019). Depletion of METTL3 inhibited the m^6^A levels of serine- and arginine-rich splicing factors (SRSF), leading to NMD of SRSF which was mediated by YTHDC1 (Li et al., 2019). Subsequently, downregulated SRSFs significantly changed alternative splicing events of several genes including BCL-X and NCOR2, contributing to suppression of GSCs self-renewal (Li et al., 2019). All these findings establish a critical role of m^6^A modifications in GSCs.

Breast cancer and ovarian cancer are common cancers in women. m^6^A modifications exert profound and diverse functions in breast cancer stem cells and ovarian cancer stem cells. In response to hypoxia, hypoxia-inducible factor (HIF)-1α and HIF-2α were stimulated to promote ALKBH5 expression in breast cancer cells; subsequently, ALKBH5 inhibited the m^6^A level in the 3′UTR of Nanog mRNA and increased NANOG expression, resulting in enhanced breast cancer stem cell phenotype (Zhang et al., 2016). Conversely, ALKBH5 knockdown in human breast cancer cells suppressed tumor initiation capacity (Zhang et al., 2016). Therefore, ALKBH5-mediated m^6^A modifications play a pivotal role in maintaining breast cancer stemness in the hypoxic environment. Aurora kinase A (AURKA) is a member of serine/threonine kinases family and was reported to stabilize METTL14 protein by preventing its ubiquitylation in breast cancer stem-like cells (Peng et al., 2021). Subsequently, upregulated METTL14 expression induced the m^6^A level of DROSHA, a Class 2 ribonuclease III enzyme, to stabilize DROSHA mRNA which was meditated by m^6^A reader IGF2BP2 (Peng et al., 2021). Intriguingly, AURKA could strengthen the binding of IGF2BP2 to DROSHA mRNA, thus promoting DROSHA expression (Peng et al., 2021). Furthermore, DROSHA interacted with β-catenin to transactivate STC1, resulting in enhanced stemness of breast cancer (Peng et al., 2021). In ovarian cancer, FTO is suggested to suppress self-renewal of ovarian CSCs. Huang et al. revealed reduced FTO expression in ovarian tumors and ovarian CSCs (Huang et al., 2020). In this study, ectopic expression of FTO in ovarian cancer cells inhibited the m^6^A level in the 3′UTR of two phosphodiesterase genes, PDE1C and PDE4B, and reduced their mRNA stability, leading to activation of second messenger 3′, 5′-cyclic adenosine monophosphate (cAMP) signaling and suppression of stemness features (Huang et al., 2020). Furthermore, FTO could suppress self-renewal of ovarian CSCs *in vivo* in an m^6^A-dependent manner (Huang et al., 2020). All these studies unveil a key role of m^6^A modifications in regulating stemness phenotype of breast cancer and ovarian cancer.

### N^6^-Methyladenosine and Hematological Tumors

Hematologic malignancies comprise three main types: leukemia, lymphoma, and multiple myeloma (MM). In acute myeloid leukemia (AML), a subpopulation of AML cells, called leukemia stem cells (LSCs), exert self-renewal capacity and is responsible for the maintenance of the AML phenotype. There have been numerous studies reporting the functional importance of m^6^A modifications in AML. Li et al. revealed increased expression of FTO in AML (Li et al., 2017). High FTO expression suppressed the m^6^A levels of ankyrin repeat and SOCS box protein 2 (ASB2) and retinoic acid receptor α (RARA), leading to reduced mRNA stability of these two genes (Li et al., 2017). However, future study is required to identify m^6^A readers that are responsible for stabilizing FTO target transcripts, such as ASB2 and RARA. Consequently, FTO promoted leukemogenesis and inhibited Tretinoin-induced AML cell differentiation (Li et al., 2017). Given the functional significance of FTO in AML, several FTO inhibitors have been developed. In a subsequent study, Su et al. reported that R-2-hydroxyglutarate (R-2HG), originally thought to be an oncometabolite, strongly inhibited FTO activity, thereby increasing global m^6^A modifications, resulting in reduced mRNA stability of MYC/CEBPA in R-2HG-sensitive leukemia cells (Su et al., 2018). Of note, R-2HG treatment also increased ASB2 and RARA expressions in R-2HG-sensitive cells, but not in the resistant cells (Su et al., 2018). Importantly, R-2HG exhibited a potent anti-tumor effect against leukemia with high FTO expression by targeting FTO-m^6^A-MYC/CEBPA axis (Su et al., 2018). However, whether and how R-2HG exerted its effect on cancer metabolism in leukemia was unclear. Accordingly, Qing et al. showed that R-2HG could effectively inhibit aerobic glycolysis in R-2HG-sensitive leukemia cells, but not in normal CD34^+^ hematopoietic stem/progenitor cells (Qing et al., 2021). Aerobic glycolysis, termed Warburg effect, converts glucose to lactate even without oxygen, thereby providing the energy required by the cancer cells. R-2HG inhibited FTO activity and increased the m^6^A level of phosphofructokinase platelet (PFKP) and lactate dehydrogenase B (LDHB), two critical glycolytic genes, thereby reducing their mRNA stability which was mediated by YTHDF2 (Qing et al., 2021). Notably, FTO, PFKP, or LDHB depletion recapitulated R-2HG-induced glycolytic inhibition and suppressed leukemogenesis *in vivo* (Qing et al., 2021). Using structure-based rational design, Huang et al. recently developed two FTO inhibitors, FB23 and FB23-2 (derivatives of meclofenamic acid), which could directly bind to FTO and suppress its demethylase activity (Huang et al., 2019). FB23-2 strongly inhibited cell proliferation but induced differentiation/apoptosis of human AML cells both *in vitro* and *in vivo*; moreover, FB23-2 exhibited a promising therapeutic efficacy in patient-derived xeno-transplantation AML mouse model (Huang et al., 2019). Notably, FB23-2 treatment could significantly eliminate LSCs in these mice models, thereby disrupting AML maintenance (Huang et al., 2019). However, the half-maximal inhibitory concentration (IC50) values of FB23 and FB23-2 in suppressing AML are still high: >20 μM and >1 μM for FB23 and FB23-2, respectively (Huang et al., 2019). To develop efficacious inhibitors against FTO, Chen’s team conducted a structure-based virtual screening of the 260,000 compounds and validation assays, leading to the identification of two compounds, CS1 and CS2, which displayed strong inhibitory effects against FTO activity and AML cell viability with 10- to 30-fold lower IC50 (Su et al., 2020). FTO was frequently overexpressed in LSCs, and pharmacological inhibition of FTO by CS1 and CS2 suppressed self-renewal of LSCs (Su et al., 2020). In addition, targeting FTO decreased the expression of immune checkpoints, such as PD-L1, PD-L2, and LILRB, to reverse immune evasion of leukemia cells (Su et al., 2020), highlighting the potential of FTO inhibitors for cancer therapy. Nevertheless, there remains some limitations for small-molecule FTO inhibitors, e.g., toxic side effects, the sensitivity and specificity of inhibitors against LSCs. As such, Cao et al. developed FTO inhibitor-loaded GSH-bioimprinted nanocomposites (GNPIPP12MA) of synergistic FTO inhibition and GSH depletion (Cao et al., 2022). Notably, GNPIPP12MA not only selectively targeted LSCs but also enhanced the efficacy of the PD-L1 blockade, thereby suppressing leukemogenesis (Cao et al., 2022). Other m^6^A regulators, such as METTL3 (Barbieri et al., 2017; Vu et al., 2017), METTL14 (Weng et al., 2018), YTHDF2 (Paris et al., 2019), YTHDC1 (Cheng et al., 2021; Sheng et al., 2021), and ALKBH5 (Shen et al., 2020; Wang et al., 2020), have also been demonstrated to regulate LSCs features and contribute to leukemogenesis. It is worth noting that Yankova et al. recently developed a highly potent and selective METTL3 inhibitor, named STM2457, that posed a strong effect in suppressing growth while increasing differentiation and apoptosis of AML (Yankova et al., 2021). Together, all these studies suggest that targeting m^6^A regulators is a potential therapeutic strategy against AML.

Myeloma is a blood cancer of plasma cells derived from bone marrow. Recent evidence implies a functional role of m^6^A in MM pathogenesis. Upregulated isocitrate dehydrogenase 2 (IDH2) in CD138^+^ MM cells reduced global RNA m^6^A modification through activating FTO (Song et al., 2021). The m^6^A level of WNT7B mRNA was decreased by IDH2, leading to increased WNT7B expression and subsequent activation of Wnt pathway which eventually facilitated tumorigenesis and progression of MM (Song et al., 2021). Consistently, FTO was highly expressed in plasma cells from MM patients, concomitant with decreased RNA m^6^A level (Xu et al., 2022). FTO inhibited m^6^A modifications of heat shock factor 1 (HSF1), thereby increasing its mRNA stability in a YTHDF2-dependent manner (Xu et al., 2022). Importantly, FTO-m^6^A-HSF1 promoted MM cells growth and metastasis (Xu et al., 2022). Similarly, ALKBH5 was overexpressed in MM and promoted MM tumorigenesis (Qu et al., 2022). ALKBH5 inhibited m^6^A modifications in 3′UTR of TNF receptor-associated factor 1 (TRAF1) and enhanced its mRNA stability, leading to activation of NF-κB and MAPK signaling pathways (Qu et al., 2022).

Lymphoma is cancer of lymphocytes from lymph nodes, spleen, thymus, or bone marrow. Han et al. reported that PIWI-interacting RNAs (piRNAs)-30473 upregulated WTAP and increased the global m^6^A level in diffuse large B-cell lymphoma (DLBCL) (Han et al., 2021). Hexokinase 2 (HK2) was further identified as the downstream target of piRNA-30473-WTAP-m^6^A, and upregulated HK2 by piRNA-30473 contributed to DLBCL tumorigenesis (Han et al., 2021). On the other hand, proto-oncogene MYC was found to transcriptionally activate ALKBH5 and FTO and inhibit m^6^A levels of SPI1 and PHF12 transcripts, thereby suppressing their mRNA translation which was mediated by YTHDF3 (Wu et al., 2021). Furthermore, depletion of ALKBH5 effectively reduced growth of B-cell lymphomas with deregulated MYC expression (Wu et al., 2021).

## Conclusion and Future Perspectives

To date, great efforts have been made to explore the roles of RNA m^6^A modifications in different biological processes, and improvements have been achieved to advance our understanding of m^6^A-mediated epitranscriptomic regulation and its potential as therapeutic targets for cancer patients. However, many questions remain elusive: 1) the origins and functions of m^6^A marks at different stages of human development are still largely unclear; 2) the contribution of m^6^A modifications in iPSC pluripotency should be further clarified; 3) m^6^A writers (e.g., METTL3) and erasers (e.g., FTO) both play an oncogenic role in several cancer types (e.g., AML). Thus, m^6^A regulators likely target different groups of transcripts and regulate different biological processes; 4) the position of m^6^A sites (e.g., 5ʹUTR, CDS, or 3ʹUTR) in transcripts likely influence the recognition of m^6^A and the subsequent RNA metabolism; 5) m^6^A readers could exhibit opposite functions. YTHDF2 promotes RNA degradation of m^6^A-modified mRNAs while IGF2BP1-3 stabilizes them, although they target different transcripts. Besides, more and more m^6^A readers are being discovered, adding to the complexity of m^6^A epitranscriptome; and 6) the crosstalk or competition among m^6^A writers, readers and erasers should be further explored. Although the functions of m^6^A regulators are context dependent, targeting m^6^A offers great potential for cancer treatment. Future studies on understanding the context-dependent role of m^6^A modification in cellular reprogramming and cancer stemness is of utmost importance.
